# Effects of Gum Arabic Coatings Enriched with Lemongrass Essential Oil and Pomegranate Peel Extract on Quality Maintenance of Pomegranate Whole Fruit and Arils

**DOI:** 10.3390/foods11040593

**Published:** 2022-02-18

**Authors:** Tatenda Gift Kawhena, Umezuruike Linus Opara, Olaniyi Amos Fawole

**Affiliations:** 1 Department of Horticultural Science, Faculty of AgriSciences, Stellenbosch University, Stellenbosch 7600, South Africa; 19547129@sun.ac.za; 2 SARChI Postharvest Technology Research Laboratory, Africa Institute for Postharvest Technology, Faculty of AgriSciences, Stellenbosch University, Stellenbosch 7600, South Africa; 3 UNESCO International Centre for Biotechnology, Nsukka 410001, Enugu State, Nigeria; 4 Postharvest Research Laboratory, Department of Botany and Plant Biotechnology, University of Johannesburg, Johannesburg 2006, South Africa

**Keywords:** gum arabic, *Punica granatum*, coating, decay

## Abstract

The effects of gum arabic coatings combined with lemongrass oil and/or pomegranate peel extract on freshly harvested mature ‘Wonderful’ pomegranate fruit were studied. Fruit were coated with gum arabic (GA) (1.5% *w/v*) alone or enriched with lemongrass oil (LM) (0.1% *v/v*) and/or pomegranate peel extract (PP) (1% *w/v*). Fruit were packed into standard open top ventilated cartons (dimensions: 0.40 m long, 0.30 m wide and 0.12 m high), and stored for 6 weeks at 5 ± 1 °C (90% RH). Evaluations were made every 2 weeks of cold storage and after 5 d of shelf life (20 °C and 65% RH). Fruit coated with GA + PP (4.09%) and GA + PP + LM (4.21%) coatings recorded the least cumulative weight loss compared to the uncoated control (9.87%). After 6 weeks, uncoated control and GA + PP + LM recorded the highest (24.55 mg CO_2_Kg^−1^h^−1^) and lowest (10.76 mg CO_2_Kg^−1^h^−1^) respiration rate, respectively. Coating treatments reduced the incidence of decay and treatments GA + LM + PP and GA + PP recorded the highest total flavonoid content between 2 and 6 weeks of storage. The findings suggest that GA coatings with/without LM and PP can be a beneficial postharvest treatment for ‘Wonderful’ pomegranates to reduce weight loss and decay development during cold storage.

## 1. Introduction

Pomegranate (*Punica granatum* L.) is a non-climacteric fruit with unique taste and flavor. It is grown in several tropical and sub-tropical regions, including the Mediterranean, China, India, South Africa and America [1]. Earlier studies showed that pomegranate is a rich source of polyphenolic compounds present in the three main parts of the fruit, namely, seed, juice and peel [2,3]. Polyphenolic compounds found in pomegranate include flavonoids (such as pelargonidin, delphinidin, catechin, epicatechin and quercetin), tannins (ellagitannins, ellagic acid, punicalagin, punicalin and pedunculagin) and phenolic acids (such as chlorogenic, ellagic, gallic and cinnamic acid) [4,5]. Based on these constituents, the pomegranate exhibits antimicrobial, anti-viral, anti-cancer, potent antioxidant and anti-mutagenic properties [2].

The quality factors of harvested pomegranate fruit decrease more rapidly under excessive loss of moisture from the fruit, fungal infection and physiological deterioration during storage and transit [6,7,8]. Thus, approaches to delay the loss of quality and extend shelf life are important. Some methods have been developed to reduce weight loss, polyphenol oxidase (PPO) mediated oxidation of phenols and microbial proliferation, which include the use of modified atmosphere packaging (MAP) [9,10,11], controlled atmosphere [12], fungicides [13]. For instance, Mphahlele et al. [14] reported that packing ‘Wonderful’ pomegranates in either ZOEpac polyliner bags or shrink wrapping reduced weight loss and maintained good visual appearance during 4 months of cold storage (7.5 ± 0.5 °C). Likewise, Lufu et al. [15] showed that different MAP polyliners (micro-perforated Xtend^®^, 2 mm macro-perforated high density polyethylene and 4 mm macro-perforated) minimized weight loss and the development of decay and shrivel in ‘Wonderful’ pomegranates stored at 5 °C for 84 d. Similar results were also reported when MAP technology was applied on several pomegranate cultivars including ‘Bhagwa’ [16], ‘Hicrannar’ [17] and ‘Primosole’ [18]. However, with the current trend towards sustainability of the environment and market, some technologies are categorized as unsafe, and customers prefer plastic-free packaging [19,20].

An alternative approach involves the use of ‘generally recognized as safe’ (GRAS) edible coatings to minimize deterioration of quality caused by uncontrolled weight loss [21,22]. Edible coatings form a semi-permeable barrier and limit moisture and gas exchange between the fruit and the surrounding atmosphere, resulting in reduced respiration and oxidation reaction rates [22]. Several polymers serve as the matrix in edible coating formulation which include, proteins, lipids and polysaccharides [22]. Gum arabic is a common edible polysaccharide with a GRAS status used in folk medicine and the food industry as a stabilizer and thickening agent in developing coatings and films [23,24]. Gum arabic coatings have been applied to reduce weight loss and minimize quality loss during cold storage and shelf life for fresh fruit and vegetables [25].

In recent years, novel methods in food preservation technology involve the development of coatings containing active ingredients, which reduce quality loss improve food safety and shelf life [26,27]. Various active ingredients possessing antimicrobial, antioxidant and antibrowning properties incorporated into coatings establish contact with food and inhibit the growth of microorganisms present on the surface [28,29,30]. Examples of these active ingredients include essential oils, plant extracts, enzymes, polyamines, amongst many others [31,32,33,34,35]. In particular, essential oils have been widely applied as postharvest treatments to control diseases in papaya [36], tomato [37], avocado [38], mango [39], pepper [40] and many others. When used to enrich edible coatings, essential oils such as lemongrass, oregano, thyme and cinnamon have demonstrated the efficacy to maintain microbial quality and extend storage and shelf life in several fresh food products [35,41].

Lemongrass (*Cymbopogan flexuosus*) essential oil (LM), which has a GRAS status granted by the United States Food and Drug Administration (FDA 21 CFR§182.20), has distinctly shown great potential to minimize disease incidence when used in vapor phase or in combination with edible coatings in liquid form in fresh whole or cut fruit, such as, ‘Fuji’ apples [42], pomegranate arils [43], fresh-cut pineapple [44] and strawberry [45]. These research studies proved the beneficial effects of essential oil-edible coating combinations for controlling microorganism growth during the cold storage of fresh fruit. Other active compounds, including antibrowning agents (N-acetylcysteine and glutathione) were also found to minimize microbial growth and browning on fresh fruit when combined with alginate, pectin and gellan-based coatings [46,47].

The innovative application of plant-derived extracts for food preservation is well-documented [48,49,50,51,52]. In particular, pomegranate peel extract (PP), which exhibits scavenging activity against superoxide anions, hydroxyl and peroxyl radicals, is a useful active ingredient in the preparation of edible coating formulations [2,51]. Such properties are attributed to the presence of bioactive compounds, such as phenolic acids, flavonoids and hydrolysable tannins in pomegranate peel [53]. These compounds in PP, including punicalagins, gallic acid and ellagic acid derivatives, reportedly inhibit the growth of microorganisms such as *Escherichia coli*, *Klebsiella pneumoni* and *Staphylococcus aureus* [54,55]. Furthermore, experimental data strongly support the antimicrobial activity of PP when applied to fresh food products to prevent microbial contamination. For instance, Turgut et al. [56] demonstrated the efficacy of PP to minimize loss of sensory quality in beef meatballs by inhibiting retard lipid and protein oxidation during refrigerated storage (4 ± 1 °C) for 8 d. When incorporated in chitosan (1% *w/v*) and alginate (2% *w/v*) coatings, PP improved the effectiveness to maintain the overall fruit quality by retarding loss of phytochemicals and antioxidant capacity of guava during 20-day cold storage (10 °C) [57].

Studies have shown that the application of edible coatings on pomegranate fruit stored under low temperature conditions suppressed decay weight loss and maintained overall quality [58,59]. Similarly, Kawhena et al. [60,61] demonstrated the efficacy gum arabic, sodium alginate and starch-based coatings to minimize weight loss and decay severity in ‘Wonderful’ pomegranates during cold storage and shelf life. However, to the best of our knowledge, few reports exist which show the effect of coatings enriched with active components such PP and/or LM on postharvest quality of any pomegranate fruit during storage and shelf life. Combining PP and LM as active components of coatings may potentially produce a synergistic effect against microbial proliferation and extend the storage and shelf life of pomegranate fruit. Furthermore, considering the economic importance and health benefits of pomegranate fruit, the present study had the specific objective to formulate coatings from PP and LM and investigate the efficacy to retard loss of physicochemical properties and phytochemical status and antioxidant capacity of ‘Wonderful’ pomegranate during cold storage.

## 2. Materials and Methods

### 2.1. Fruit Procurement and Handling

Pomegranate (cv. Wonderful) fruit were harvested at commercial maturity (total soluble solids = 15.4, citric acid = 1.52%) from Blydeverwacht farm, Wellington, Western Cape (33°48′0″ S, 19°53′0″ E), South Africa. Fruit with defects (sunburn, decay and cracks) were discarded and healthy whole fruit were phytosanitized by dipping in 0.02% NaClO for 5 min and allowed to dry at room temperature before storage at 5 °C (90% RH).

### 2.2. Preparation of Raw Materials and Coating Solutions

#### 2.2.1. Raw Materials

Lemongrass (*Cymbopogan flexuosus*) (Umuthi Botanicals Co. Wilderness, South Africa) essential oil was stored at 4 °C before being used in making coating solutions. Pomegranate peel extract was obtained following an extraction method outlined by Kanatt et al. [62], with slight modifications. Briefly, 100 g of pomegranate peel was continuously agitated with warm (25 °C) milli-Q water (1000 mL) for 1 h. After cooling, the extract was filtered through cheesecloth, and all filtrates were collected and centrifuged at 4000× g rpm for 15 min (4 °C). The supernatant was concentrated in a rotary evaporator (G3 Heidolph, Germany) at 40 °C, and the concentrate was frozen at −40 °C and dehydrated for 72 h in a freeze dryer (VirTis Co., Gardiner, NY, USA) at a vacuum pressure of 7 milliTorr and the condenser temperature of −88.7 °C. 

#### 2.2.2. Coating Solutions

Gum arabic (GA) (1.5% *w/v*) solutions were prepared according to Kawhena et al. [63] by dissolving 1.5 g GA (Sigma-Aldrich, Johannesburg, South Africa) coating powder in 100 mL milli-Q water and under continuous stirring at low heat (50 °C) for 90 min on a hot plate stirrer. The coating solution was filtered using cheesecloth to remove any undissolved impurities and allowed to cool to 20 °C. After that, glycerol (1% *v/v*) (plasticizing agent) and tween 80 (0.05% *v/v*) (emulsifier) were added to the coating formulation followed by LM (0.1% *v/v*) and/or PP (1% *w/v*). The pH of the final coating solution was adjusted to 5.6 with NaOH solution. The final coating solution was homogenized at 2500 rpm for 30 min in an overhead stirrer (Scientech Co., Indore, India). 

### 2.3. Experimental Design, Packaging and Storage

**Figure 1:**
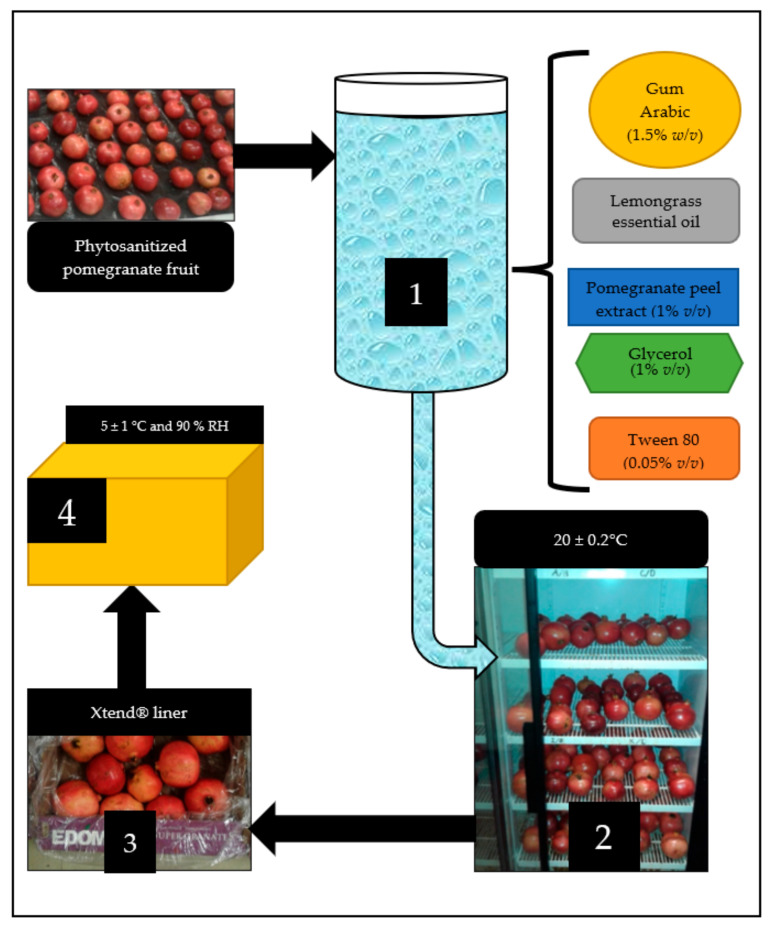
Flow diagram of the process of coating (1), drying (2), packaging (3) and cold storage (4) of phytosanitized ‘Wonderful’ pomegranate fruit.

The fruits were dipped into each coating treatment and allowed to dry at 20 ± 0.2 °C. A completely randomized design was applied for the four coating treatments (Figure 1). The fruits were packed into standard open top ventilated cartons (dimensions: 0.40 m long, 0.30 m wide and 0.12 m high) (10 fruit per carton) containing micro-perforated (20 µm) Xtend^®^ liners as used in the commercial postharvest handling of pomegranates. All fruit were stored (5 ± 1 °C and 90% RH) for 42 d. Sampling for analysis of whole fruit physical properties and aril juice chemical attributes, phytochemicals and antioxidant properties was randomly conducted at 2-weeks intervals during cold storage and after 5 d of shelf life (20 ± 0.2 °C). After sanitization and application of coatings, measurements for each treatment were taken for all parameters investigated prior to cold storage.

### 2.4. Physiological Response

#### 2.4.1. Weight Loss

Weights loss was determined continuously from 10 randomly selected fruit per treatment at every 2-week interval throughout cold storage (5 ± 1 °C and 90% RH) using an electronic weighing balance (ML3002.E, Mettler Toledo, Switzerland) [64]. Weight loss was determined using Equation (1):

W_L_ = (W_O_ − W_L_)/W_O_ × 100 (1)

where W_L_ weight loss (%) is, W_O_ is the initial weight (g) of fruit and W_L_ is the fruit weight (g) at the time of analysis.

#### 2.4.2. Respiration Rate

Respiration rate was determined at 2-weeks intervals cold storage (5 ± 1 °C and 90% RH) followed by 5 d of shelf life (20 ± 0.2 °C) based on CO_2_ evolution and fresh fruit weight in a closed system according to the technique reported by Caleb et al. [65] using a gas analyzer with an accuracy of ±0.5% (Checkmate 3, PBI Dansensor, Ringstead, Denmark). In triplicates per treatment combination, three fruit were placed in a 3 L hermetically sealed glass jar for 2 h with a lid containing a rubber septum in the middle. The CO_2_ production within the glass jar was measured from the headspace through the rubber septum. The device was auto-calibrated with the atmospheric gas composition, and the results were expressed as a percentage of gases.

### 2.5. Peel Microstructure Modification

Scanning electron microscope (Angstrom Scientific Inc., Cambridge, England) was used to observe the changes in the peel morphology and determine the thickness of coating solution applied on peel [66]. Prior to the SEM observation, the pomegranate samples were fixed and coated with a fine gold layer (40–50 nm), mounted onto aluminum stubs, and photographed. All samples were examined using an accelerating voltage of 5 kV. 

### 2.6. Fruit External and Internal Decay

External decay was determined by visual assessment of fruit for fungi development in triplicates of 30 fruit (1 replicate = 30 fruit = 3 cartons) per treatment [67]. Internal decay was assessed in triplicates of 30 fruit (1 replicate = 30 fruit = 3 cartons) by carefully cutting fruit open with sterilized knives along the equatorial axis and carefully inspecting arils for decay. The results were expressed as a percentage of the total fruit assessed.

### 2.7. Physicochemical Properties

#### 2.7.1. Color

Fruit peel color was determined from 10 randomly selected fruit per treatment along the equatorial axis of each fruit at two opposite spots and recorded in CIELAB coordinates (L*, a*, b*) using a pre-calibrated Minolta Chroma Meter CR-400 (Minolta Corp., Osaka, Japan) [68,69]. The color parameters Chroma and hue angle were calculated using Equations (2) and (3):

Chroma (C*) = (a*^2^ + b*^2^)^1/2^ (2)

Hue angle (h°) = arctan (b*/a*)(3)

#### 2.7.2. Total Soluble Solids and Titratable Acidity

Pomegranate juice (PJ) was extracted in triplicates from 10 fruit per treatment (10 fruit = 1 replicate) by carefully cutting each fruit at the equatorial zone with sharpened knives (Sigma-Aldrich, Johannesburg, South Africa) and manually separating arils. A blender (Mellerware, South Africa) was used to extract pomegranate juice (PJ) from arils (without crushing the kernels). In triplicates, titratable acidity (TA) was evaluated by diluting PJ (2 mL) with milli-Q water (70 mL) and titrated with 0.1 N NaOH to an endpoint of pH = 8.2 using a Metrohm 862 compact auto titrosampler (Herisau, Switzerland) [64]. The results were expressed as percentage citric acid equivalents (% CAE). Total soluble solids (TSS) were determined using a digital refractometer (Atago, Tokyo, Japan), and the results were expressed as degree Brix (°Brix). The TSS/TA ratio and BrimA index were calculated to understand further the relationship between TSS and TA. BrimA index was calculated using TSS − k × TA, where k is the tongue’s sensitivity index assigned a value of 2 to avoid negative BrimA index [68,70]. 

### 2.8. Phytochemicals and Antioxidant Activity

#### 2.8.1. Phytochemicals

Total phenolic (TP) content of PJ was evaluated according to the Folin–Ciocalteu microplate method outlined by Horszwald and Andlauer [71] with slight modifications. Briefly, 100 µL of 10% Folin-C method were added to 20 µL of 6-fold diluted PJ in 96-well microplate reader and incubated for 3 min at room temperature. Subsequently, 80 µL of sodium carbonate (7.5% *w/v*) were added to the solution and heated for 1 h at 30 °C in oven. The absorbance of the solution was read at 750 nm, and results were expressed in mg gallic acid equivalent (GAE) per liter (mg GAE/LPJ).

Total flavonoids (TF) of PJ were determined using the method outlined by Herald et al. [72] with slight modifications. Briefly, PJ samples (25 µL) were mixed with 5% sodium nitrite solution (10 µL), and subsequently with aluminum chloride (10%, 15 µL), sodium hydroxide (1 M, 50 µL) and distilled water (100 µL). The absorbance of the mixture was measured spectrophotometrically in a 96-well microplate reader at 517 nm. Catechin was used as a standard at 5–250 µg/mL to generate a calibration curve (R^2^ = 0.9963), and results were expressed in mg catechin equivalents (CE) per liter.

A modified pH differential method outlined by Mphahlele et al. [73] was used to determine the total monomeric anthocyanin concentration of PJ. Briefly, in triplicates, supernatant (150 µL) from extracted PJ was diluted with 1mL of potassium chloride buffer (pH = 1.0) and sodium acetate buffer (pH = 4.5), separately and the absorbance was determined spectrophotometrically at 510 nm and 700 nm. The results were expressed as milligram cyanidin-3-glucoside equivalents per liter of PJ (mgC3g E/L PJ) according to Equations (4) and (5),

A = (A_510_ − A_700_) pH_1.0_ − (A_510_ − A_700_) pH_4.5_ (4)

Total anthocyanins (mgL^−1^) = [A × MW × DF)/∈] (5)

where A is absorbance values at 510 nm and 700 nm, Є = Cyanidin-3-glucoside molar absorbance (26,900), MW = Cyanidin-3-glucoside molecular weight (449.2 g/mol), DF = Dilution factor, L = Cell path length (1 cm).

Ascorbic acid of PJ was determined according to the method outlined by Munhuweyi et al. [74], adapted to a microplate method. Briefly, 9 mL of 1% metaphosphoric acid (MPA) was added to 1 mL of PJ at room temperature and vortexed for 30 s. The solution was sonicated for 5 min and centrifuged at 40,000 rpm for 20 min at 4 °C. The supernatant was collected and further diluted with 1% MPA. Subsequently, 20 µL of 20-fold diluted supernatant was then added to 180 µL of 2.6-dichlorophenolindophenol dye (0.025%), and the solution was incubated for 20 min in the dark. Ascorbic acid content of PJ was measured spectrophotometrically in a 96-well microplate reader at 517 nm and extrapolated from the calibration curve of standard L-ascorbic acid (0.2–1 mg/mL) with R^2^ > 0.90, and the results were expressed as ascorbic acid units per liter of PJ (µg AAE/LPJ).

#### 2.8.2. Antioxidant Activity

A microplate method was used to determine free radical scavenging activity (RSA) of PJ spectrophotometrically according to the procedure described by Horszwald and Andlauer [71] with slight modifications. In triplicates, 200 µL of 1,1-diphenyl-2-picryl-hydrazyl (DPPH) working solution was added to 100 µL of the 6-fold diluted sample, and after 5 min, absorbance of samples, standards and blanks were determined spectrophotometrically at 520 nm using a 96-well microplate reader. Final test absorbency was calculated according to the following Equation (6):

Test absorbency = blank absorbency − (test absorbency − color correction absorbency)(6)

The RSA of juice was expressed as ascorbic acid (millimoles) equivalent per liter of pomegranate juice (mM AAE/LPJ). 

Ferric reducing antioxidant power assay of PJ was done following the method described by De La Torre et al. [75] using ferric reducing antioxidant power (FRAP) solution prepared from acetate buffer (300 mM acetate buffer, pH = 3.6), 40 mM of 2,4,6-Tri(2-pyridyl)-s-triazine (TPTZ) solution and 20 mM of FeCl_3_ solution. Briefly, 200 µL of FRAP working solution was added to 25 µL of 6-fold diluted PJ extract, and the solution was vortexed for 30 s. Then, the solution was incubated at 37 °C for 30 min, and absorbance was determined spectrophotometrically in a 96-well microplate reader at 517 nm. Trolox (100–1000 µM) standard curve was used to extrapolate values, and the results were expressed as Trolox (µM) equivalents per liter of PJ (µM TE/LPJ).

Antioxidant activity of PJ was also assessed with an 2,2′-azinobis (3-ethylbenzothiazoline-6-sulfonic acid) (ABTS^+^) assay according to the protocol reported by Chirinos et al. [76] using ABTS^+^ working solution prepared from ABTS (2.6 mM) and potassium persulphate (2.6 mM). Briefly, 200 µL of ABTS+ working solution in triplicates was added to 15 µL of PJ sample extract into wells. The solution was shaken and incubated at 37 °C in the dark for 30 min for measurement. The absorbance was evaluated spectrophotometrically at 750 nm. Antioxidant activity was measured as the µmol of Trolox equivalents (TE) per liter sample from a Trolox standard curve (µmol TE/LPJ).

### 2.9. Statistical Analysis

The results of all studied parameters are presented as mean (±SE) values. Experimental data were subjected to two-way analysis of variance (ANOVA) at 95% confidence interval using SAS Software (SAS Enterprise Guideline 7.1, Carey, NC, USA). Least significant differences (LSD) were calculated according to Fisher’s least significant difference test to compare differences between means at a 5% significance level. Graphical presentations were made using GraphPad Prism software version 8.4.3 (GraphPad Software, Inc., San Diego, CA, USA).

## 3. Results

### 3.1. Physiological Response

#### 3.1.1. Weight Loss and Peel Microstructure

**Figure 2:**
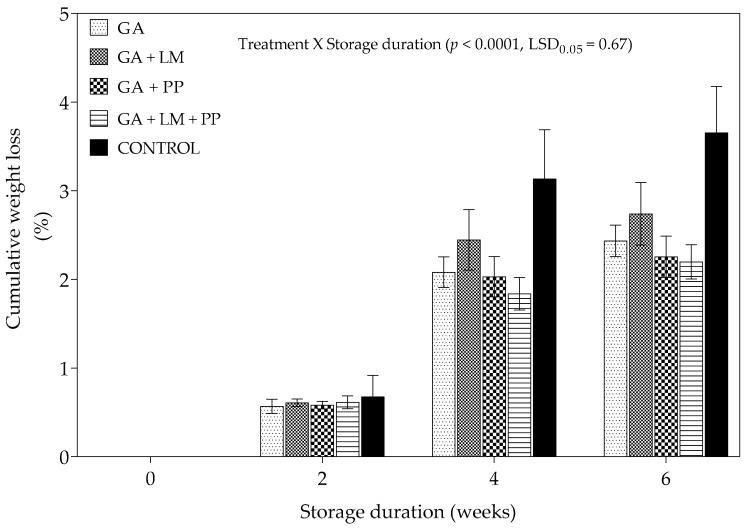
Cumulative weight loss (%) of ‘Wonderful’ pomegranate whole fruit coated with gum arabic (GA) coatings enriched with lemongrass oil (LM) (0.1% *v/v*) and/or pomegranate peel extract (PP) (1% *w/v*) and stored at 5 ± 1 °C (95 ± 2% RH) for 6 weeks followed by 5 d at shelf-life conditions (20 ± 1 °C and 60% RH). Vertical bars represent the standard error (SE) of mean values of 10 measurements (10 fruit per treatment). LSD_0.05_ represents least significant difference (*p* < 0.05).

**Figure 3:**
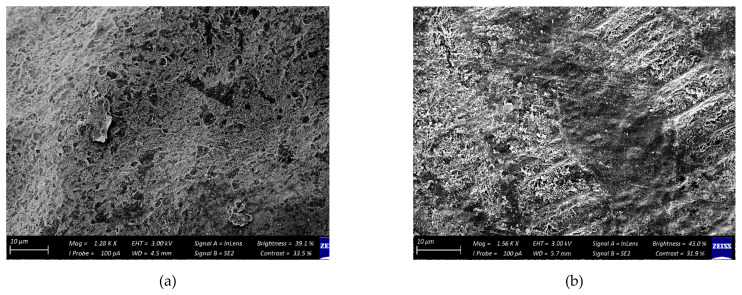

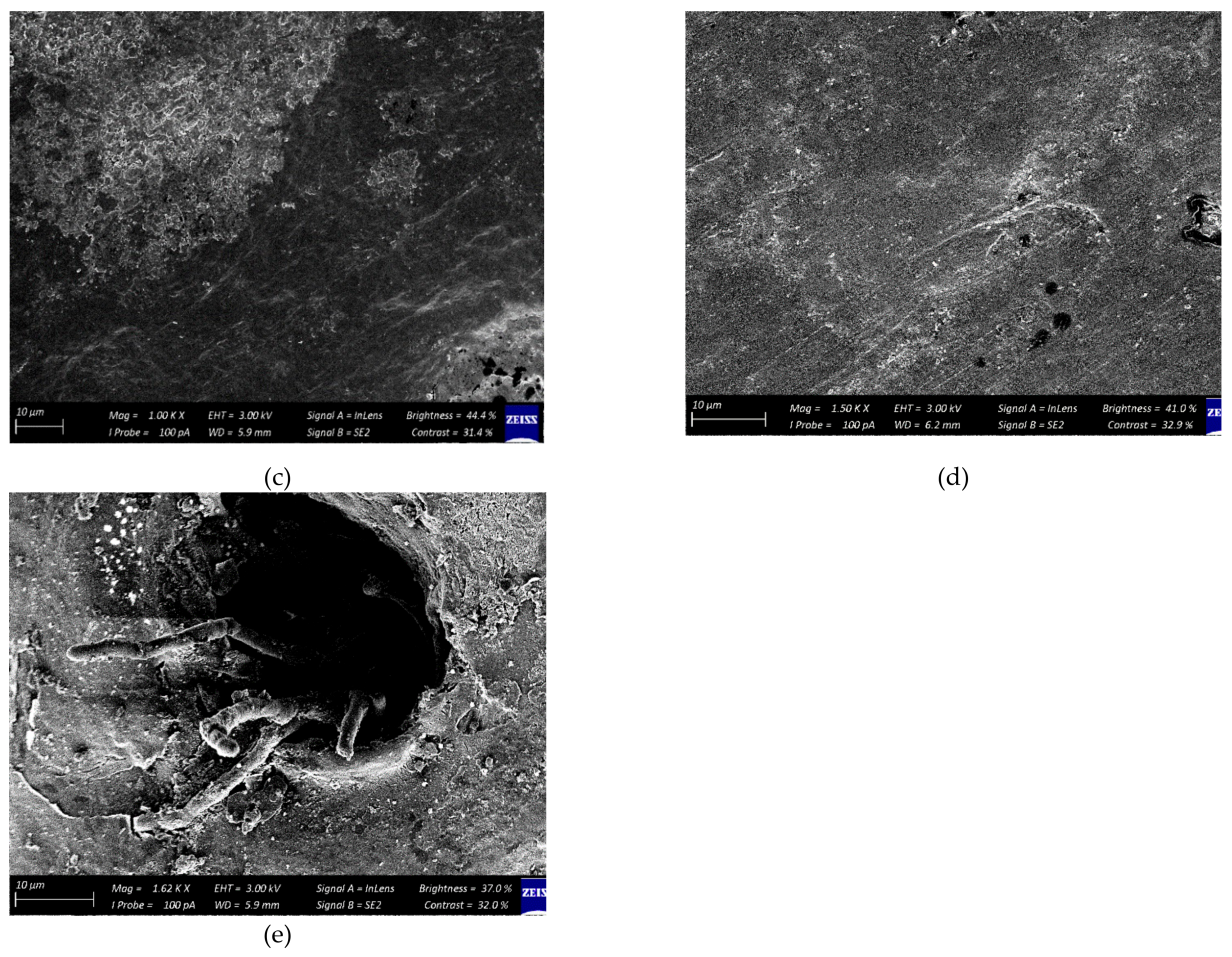
Scanning electron micrographs of ‘Wonderful’ pomegranate peel after 6 weeks of cold storage at 5 °C. (**a**)—gum arabic (GA); (**b**)—gum arabic + lemongrass oil (GA + LM); (**c**)—gum arabic + pomegranate peel extract (GA + PP); (**d**)—gum arabic + lemongrass oil + pomegranate peel extract (GA + LM + PP); (**e**)—Uncoated control.

Figure 2 shows that weight loss steadily increased from day 0 to 6 weeks of storage across all treatments. However, cumulative weight loss was lesser for fruit coated with GA (with or without LM and/or PP) coatings when compared to uncoated fruit. After the 6-week storage period, the lowest (2.20%) and highest (3.65%) cumulative weight loss were recorded for fruit coated with GA + LM + PP and uncoated fruit (packaged in Xtend^®^ liners), respectively. Therefore, GA coatings enriched with LM and PP (GA + LM + PP) proved to be the most effective treatment in reducing weight loss. Figure 2 shows scanning electron micrographs for coated and uncoated pomegranate peels after 6 weeks of cold storage. The cross-section image of the coated pomegranate peels (Figure 3a–d) demonstrated that coating solution clearly covered the peel with a coating thickness of approximately 5 µm. The images showed smooth surfaces with some pores or cracks and a compact structure on all coated pomegranate peels. On the contrary, the uncoated control had more open lenticels on the peel surface (Figure 3e). 

#### 3.1.2. Respiration Rate

**Figure 4:**
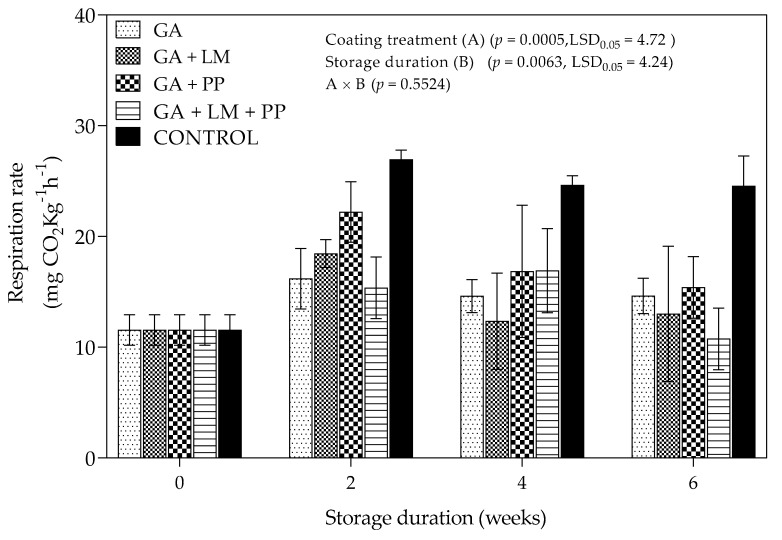
Respiration rate (mg CO_2_Kg^−1^h^−1^) of ‘Wonderful’ pomegranate fruit coated with gum arabic (GA) coatings enriched with lemongrass oil (LM) (0.1% *v/v*) and/or pomegranate peel extract (PP) (1% *w/v*) and stored at 5 ± 1 °C (95 ± 2% RH) for 6 weeks followed by 5 d at shelf-life conditions (20 ± 1 °C and 60% RH). Vertical bars represent the standard error (SE) of mean value of three replicates (3 fruit per replicate). LSD_0.05_ represents least significant difference (*p* < 0.05).

Figure 4 indicates the change in the respiration rate (R_CO2_) of ‘Wonderful’ pomegranates subjected to various treatments under cold storage followed by shelf-life conditions. At every storage period, uncoated control fruit recorded the highest R_CO2_ compared to coated fruit. There was an initial increase in R_CO2_ from day 0 to 2-week storage period uncoated fruit and treatments GA, GA + LM and GA + PP. However, the increase was greater for uncoated fruit than coated fruit (GA, GA + LM and GA + PP). Subsequently, there was a decrease in R_CO2_ across uncoated fruit and the coating treatments (GA and GA + PP). After the 6-week storage period, the highest (24.55 mg CO_2_Kg^−1^h^−1^) and lowest (10.76 mg CO_2_Kg^−1^h^−1^) R_CO2_ were recorded for uncoated fruit and treatment GA + PP + LM, respectively. Overall, the application of GA coatings with/without LM and/or PP reduced R_CO2_ compared to uncoated fruit during storage.

### 3.2. Decay Incidence and Fruit Internal Decay

**Figure 5:**
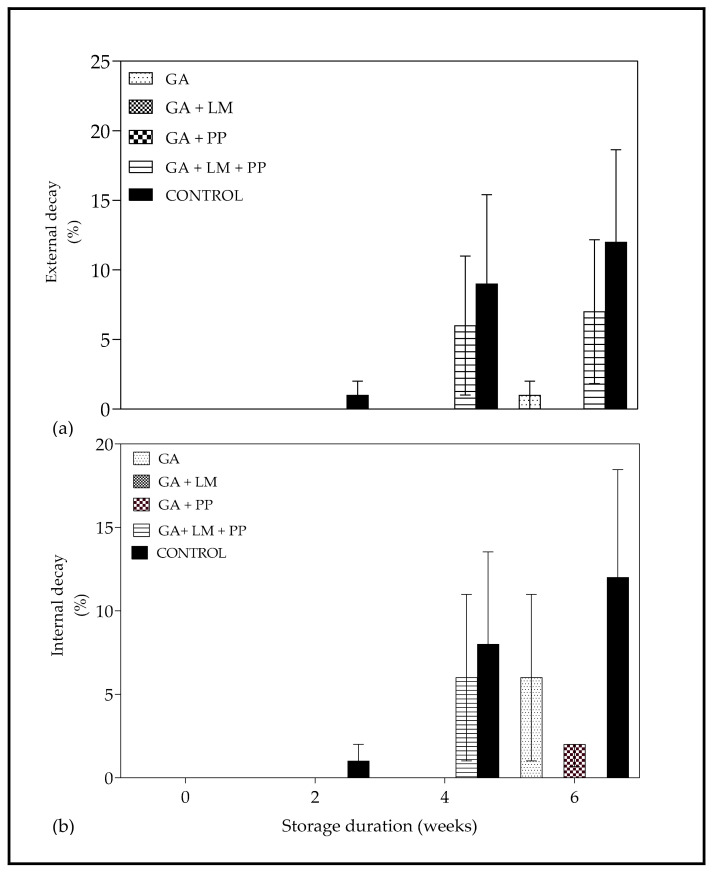
(**a**) External decay (%) and (**b**) internal decay of ‘Wonderful’ pomegranate whole fruit coated with gum arabic (GA) coatings enriched with lemongrass oil (LM) (0.1% *v/v*) and/or pomegranate peel extract (PP) (1% *w/v*), and stored at 5 ± 1 °C (95 ± 2% RH) for 6 weeks. Vertical bars represent the standard error (SE) of mean values calculated from 10 measurements [1 carton (10 fruit) = 1 measurement].

External decay was first observed after 2 weeks of cold storage with uncoated fruit showing 1% decay incidence (Figure 5a). Decay incidence increased as storage duration was extended and after 6 weeks, uncoated fruit recorded 12% decay incidence. Moreover, fruit coated with GA + LM and GA + PP recorded 0% decay incidence after 6 weeks of cold storage. Similar to external decay, internal decay was first observed after 2 weeks with uncoated control fruit recording 1% decay incidence (Figure 5b). Internal decay increased with storage duration and after 6 weeks, uncoated control fruit recorded 12% decay incidence. No internal decay was observed for fruit coated with GA, GA + LM and GA + PP throughout the 6 weeks of cold storage.

### 3.3. Physicochemical and Textural Properties

#### 3.3.1. Color

**Figure 6:**
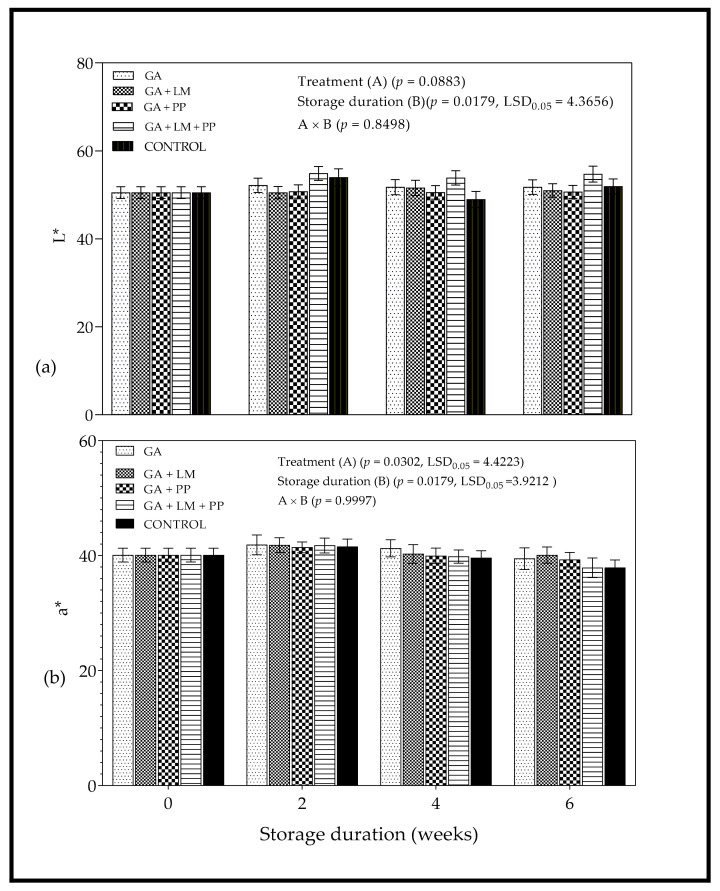
Color attributes (**a**) lightness (L*) and (**b**) redness (a*) of ‘Wonderful’ pomegranate whole fruit coated with gum arabic (GA) coatings enriched with lemongrass oil (LM) (0.1% *v/v*) and/or pomegranate peel extract (PP) (0.1% *w/v*) and stored at 5 ± 1 °C (95 ± 2% RH) for 6 weeks. Vertical bars represent the standard error (SE) of mean value calculated from 10 measurements per treatment (1 fruit = 1 measurement). LSD_0.05_ represents least significant difference (*p* < 0.05).

**Figure 7:**
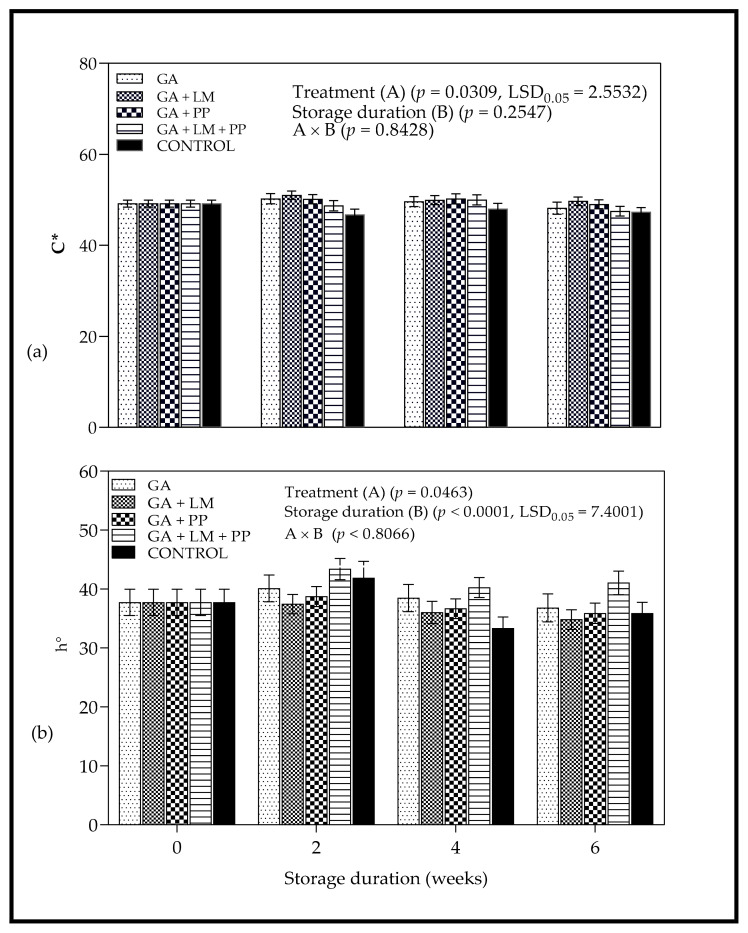
Color attributes, (**a**) Chroma (C*) and (**b**) hue angle (h°) of ‘Wonderful’ pomegranate whole fruit coated with gum arabic (GA) coatings enriched with lemongrass oil (LM) (0.1% *v/v*) and/or pomegranate peel extract (PP) (0.1% *w/v*) and stored at 5 ± 1 °C (95 ± 2 % RH) for 6 weeks followed by 5 d at shelf-life conditions (20 ± 1 °C and 60%RH). Vertical bars represent the standard error (SE) of mean value calculated from 10 measurements per treatment (1 fruit = 1 measurement). LSD_0.05_ represents least significant difference (*p* < 0.05).

Figure 6 shows the changes in color attributes (L*, a*, C* and h°) of uncoated and coated ‘Wonderful’ pomegranates as a function of storage time. Despite slight changes in lightness (L*) with the passage of storage time, statistical analysis showed no significant change in L* (Figure 6a) and redness (a*) (Figure 6b). Slight differences in color saturation (C*) between coated and uncoated fruit were only observed after the 4-week storage period (Figure 7a), where coated fruit (GA + LM = 51.03 ± 0.93) had higher color saturation compared to untreated control (46.74 ± 1.22); also, higher a* values were observed compared to untreated control. Upon application of coating treatments on day 0, GA + LM coated fruit recorded higher h° values compared to the other treatments. Despite some fluctuations, there were no significant changes in h° with the extension of storage duration for most of the coating treatments (Figure 7b).

#### 3.3.2. Total Soluble Solids and Titratable Acidity

**Figure 8:**
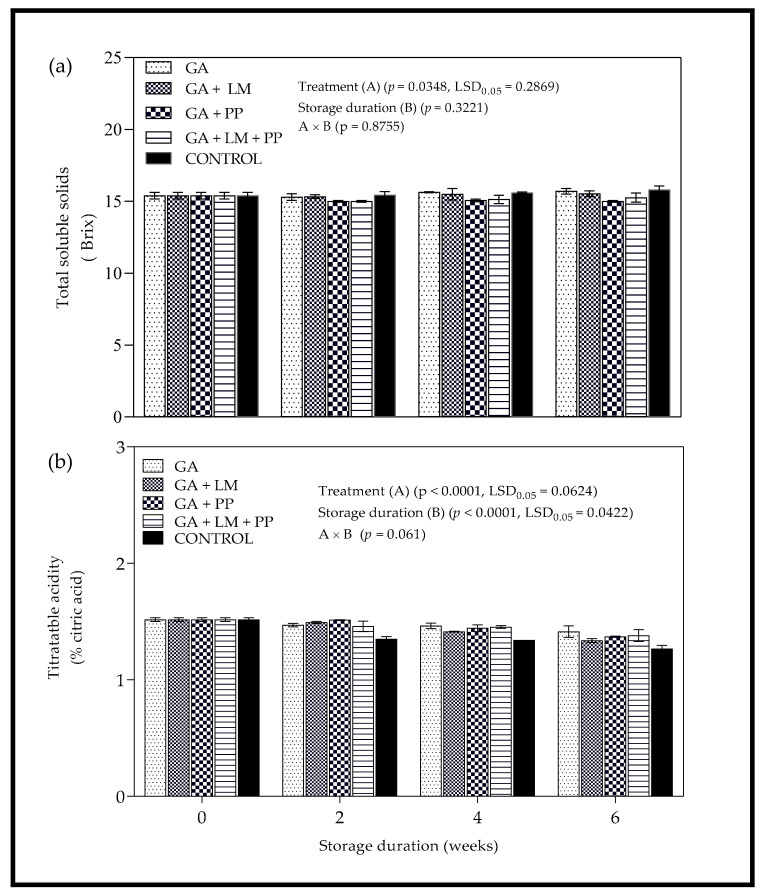
(**a**) Total soluble solids (°Brix) and (**b**) titratable acidity (% citric acid equivalents) of ‘Wonderful’ pomegranate whole fruit coated with gum arabic (GA) coatings enriched with lemongrass oil (LM) (0.1% *v/v*) and/or pomegranate peel extract (PP) (1% *w/v*) and stored at 5 ± 1 °C (95 ± 2% RH) for 6 weeks followed by 5 d at shelf-life conditions (20 ± 1 °C and 60% RH). Vertical bars represent the standard error (SE) of mean values calculated from three measurements (Homogenized aril juice extracted from 10 fruit = 1 replicate). LSD_0.05_ represent least significant difference (*p* < 0.05).

Figure 8a shows the effect of coatings and storage duration on TSS of aril juice from ‘Wonderful’ pomegranates. The TSS content was significantly (*p* = 0.0348) influenced by coating treatments. Overall, there was an increase in TSS from day 0 to 6 weeks, with uncoated fruit recording higher values than all coated treatments. After the 6-week storage period, highest (15.80 °Brix) and lowest (15.00 °Brix) TSS values were recorded for uncoated fruit and GA + PP. However, there was no significant difference between TSS content of GA coated fruit at the same period. For coating treatments GA + PP and GA + LM + PP, TSS content of aril juice mostly declined from day 0 to 6 weeks. Figure 8b shows the evolution of TA of ‘Wonderful’ pomegranate juice extracted from coated and uncoated fruit during cold storage. There was a decrease in TA content across all treatments as storage period was extended from day 0 to 6 weeks. However, the rate of decrease was higher in uncoated fruit than in all coating treatments. After the 6-week storage period, GA (1.41% CAE) and uncoated fruit (1.27% CAE) recorded the highest and lowest TA content, respectively. 

#### 3.3.3. TSS/TA and Brim A

**Figure 9:**
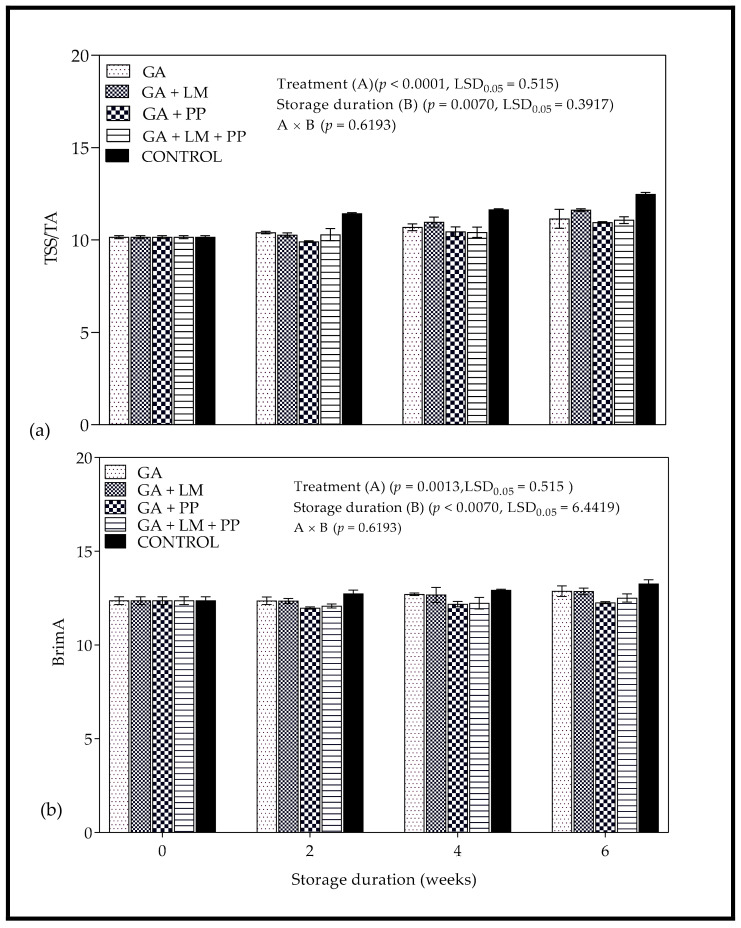
(**a**) TSS/TA and (**b**) BrimA of ‘Wonderful’ pomegranate whole fruit coated with gum arabic (GA) coatings enriched with lemongrass oil (LM) (0.1% *v/v*) and/pomegranate peel extract (PP) (1% *w/v*) and stored at 5 ± 1 °C (95 ± 2% RH) for 6 weeks followed by 5 d at shelf-life conditions (20 ± 1 °C and 60% RH). Vertical bars represent the standard error (SE) of mean values calculated from three measurements (Aril juice extracted from 10 fruit = 1 replicate = 1 measurement). LSD_0.05_ represent least significant difference (*p* < 0.05).

As shown in Figure 9a, TSS/TA increases as storage duration was extended, with coating treatments recording lower values than control. Statistical analysis also showed that the main differences in BrimA index were due to interactions (*p* < 0.0107) between coating treatments and storage duration. The changes in Brim A values as shown in Figure 9b reflected the levels of TSS and TA in fruit during storage.

### 3.4. Phytochemicals

#### 3.4.1. Total Phenolic Content

**Figure 10:**
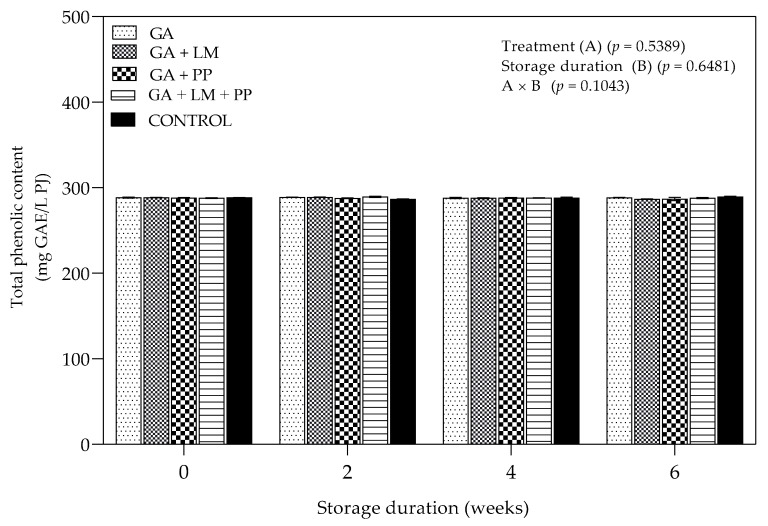
Total phenolic content (mg GAE/L PJ) of ‘Wonderful’ pomegranate whole fruit coated with gum arabic (GA) coatings enriched with lemongrass oil (LM) (0.1% *v/v*) and/pomegranate peel extract (PP) (1% *w/v*) and stored at 5 ± 1 °C (95 ± 2% RH) for 6 weeks followed by 5 d at shelf-life conditions (20 ± 1 °C and 60% RH). Vertical bars represent the standard error (SE) of mean values calculated from three measurements (Aril juice extracted from 10 fruit = 1 replicate = 1 measurement). LSD_0.05_ represent least significant difference (*p* < 0.05).

Figure 10 shows the concentration of total phenolic content (TPC) during cold storage of ‘Wonderful’ pomegranates. As storage was extended, there were no significant changes in TPC across all coating treatments. After the 6-week storage period, GA + LM and uncoated fruit recorded the lowest (286.583 mg GAE/L PJ) and highest (289.24 mg GAE/L PJ) TPC, respectively. 

#### 3.4.2. Total Anthocyanin Content

**Figure 11:**
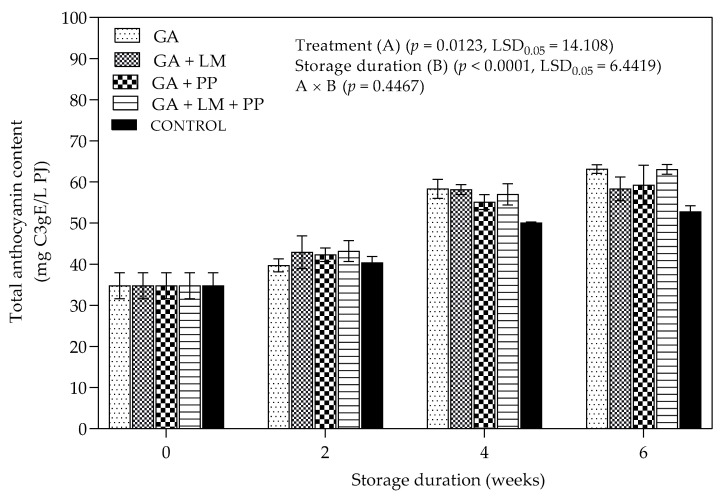
Total anthocyanin content (mg C3gE/L PJ) of ‘Wonderful’ pomegranate whole fruit coated with gum arabic (GA) coatings enriched with lemongrass oil (LM) (0.1% *v/v*) and/pomegranate peel extract (PP) (1% *w/v*) and stored at 5 ± 1 °C (95 ± 2% RH) for 6 weeks followed by 5 d at shelf-life conditions (20 ± 1 °C and 60% RH). Vertical bars represent the standard error (SE) of mean values calculated from three measurements (Aril juice extracted from 10 fruit = 1 replicate = 1 measurement). LSD_0.05_ represent least significant difference (*p* < 0.05).

The change in total anthocyanin content (TAC) was evaluated for all treatments over 6 weeks of cold storage and 5 d under shelf-life conditions (Figure 11). There was an increase in TAC for coated and uncoated fruit from day 0 to 6 weeks. However, the rate of increase was higher in coating treatments (GA, GA + LM and GA + PP) than uncoated fruit. At the end of the storage period, the highest (63.14 mg C3G/L PJ) and lowest (52.77 mg C3G/L PJ) TAC values were recorded for GA and uncoated fruit. Overall, coating treatments (GA, GA + LM and GA + PP) recorded significantly higher TAC than uncoated fruit between 4 and 6 weeks of storage.

#### 3.4.3. Total Flavonoid Content 

**Figure 12:**
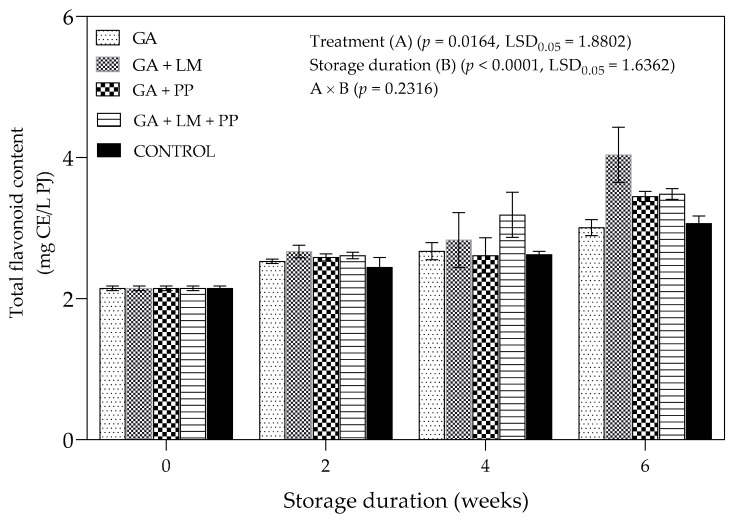
Total flavonoid content (mg CE/L PJ) of ‘Wonderful’ pomegranate whole fruit coated with gum arabic (GA) coatings enriched with lemongrass oil (LM) (0.1% *v/v*) and/pomegranate peel extract (PP) (1% *w/v*) and stored at 5 ± 1 °C (95 ± 2% RH) for 6 weeks followed by 5 d at shelf-life conditions (20 ± 1 °C and 60% RH). Vertical bars represent the standard error (SE) of mean values calculated from three measurements (Aril juice extracted from 10 fruit = 1 replicate = 1 measurement). LSD_0.05_ represent least significant difference (*p* < 0.05).

Figure 12 describes the overall effect of coating treatments on total flavonoid (TF) content of aril juice from ‘Wonderful’ pomegranates. There was an increase in the TF content as storage duration was extended for coated and uncoated fruit. However, the rate of increase was higher for coating treatments than for uncoated fruit. At the end of the storage period, the highest (4.04 mg CE/L PJ) and lowest (3.01 mg CE/L PJ) and TF content was recorded for GA + PP and uncoated fruit.

#### 3.4.4. Ascorbic Acid Content

**Figure 13:**
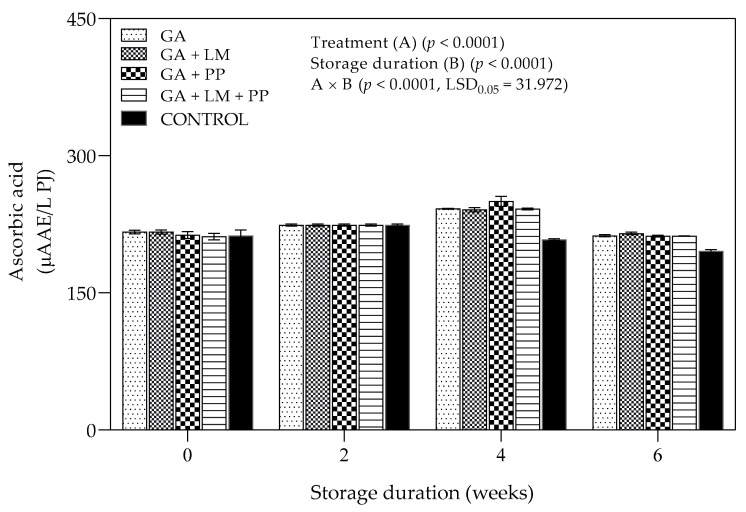
Ascorbic acid (µAAE/L PJ) of ‘Wonderful’ pomegranate whole fruit coated with gum arabic (GA) coatings enriched with lemongrass oil (LM) (0.1% *v/v*) and/pomegranate peel extract (PP) (1% *w/v*) and stored at 5 ± 1 °C (95 ± 2% RH) for 6 weeks followed by 5 d at shelf-life conditions (20 ± 1 °C and 60% RH). Vertical bars represent the standard error (SE) of mean values calculated from three measurements (Aril juice extracted from 10 fruit = 1 replicate = 1 measurement). LSD_0.05_ represent least significant difference (*p* < 0.05).

Statistical analysis showed a significant interaction (*p* = 0.03) between coating treatment and storage duration on ascorbic acid content; storage duration factor had the highest contribution (*p* < 0.0001) to the interaction compared to the coating treatments (*p* = 0.9226) (Figure 13). There was an increasing pattern of change in ascorbic acid content for coating treatments from day 0 to 4 weeks, followed by a decrease until the end of storage. However, in uncoated fruit, ascorbic acid increased from day 0 to 4 weeks, followed by a decrease until the 6-week storage period. Furthermore, coating treatments recorded significantly higher than uncoated fruit after 4 and 6 week-storage periods. After 6 weeks, the highest (212.49 µAAE/L PJ) and lowest (195.71 µAAE/L PJ) ascorbic acid content was recorded for GA and uncoated fruit, respectively.

### 3.5. Antioxidant Properties

#### 3.5.1. DPPH Radical Scavenging Activity

**Figure 14:**
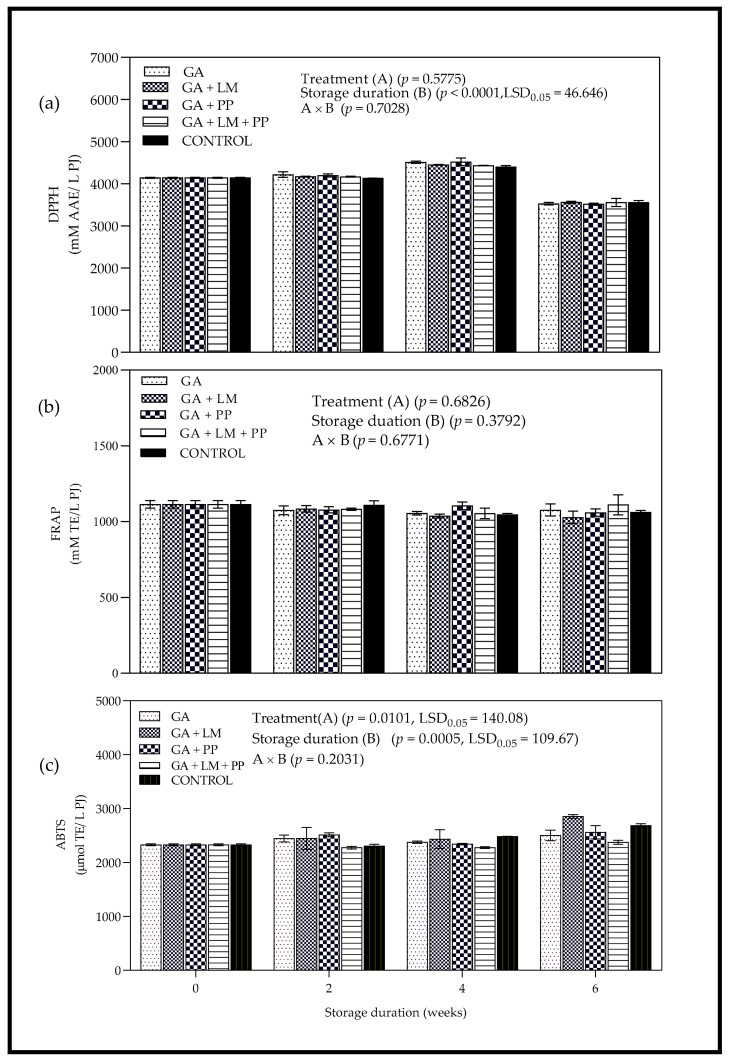
Antioxidant properties determined by (**a**) DPPH (mM AAE/L PJ), (**b**) FRAP (mM TE/L PJ) and (**c**) ABTS⁺ (µmol TE/L PJ) of ‘Wonderful’ pomegranate whole fruit coated with gum arabic (GA) coatings enriched with lemongrass oil (LM) (0.1% *v/v*) and/pomegranate peel extract (PP) (1% *w/v*) and stored at 5 ± 1 °C (95 ± 2% RH) for 6 weeks. Vertical bars represent the standard error (SE) of mean values calculated from three measurements. LSD_0.05_ represents least significant difference (*p* < 0.05).

Analysis of variance showed marked differences among coating treatments as storage duration was extended; interaction of coating treatments and storage duration had a significant (*p* < 0.0001) effect on RSA as determined by the DPPH assay (Figure 14a). Application of GA (GA, GA + PP) coatings enhanced the RSA of PJ, after 4 and 6-week storage periods, respectively. However, there were no marked differences in RSA values between uncoated and coated fruit as storage duration was extended from day 0 to 6 weeks. After the 6-week storage period, the lowest values of RSA were recorded for both uncoated and coated fruit. Furthermore, treatments GA + LM (3559.72 mM AAE/L PJ) and GA + PP (3513.97 mM AAE/L PJ) recorded the highest and lowest RSA values as determined by the DPPH assay.

#### 3.5.2. Ferric Reducing Antioxidant Power

Figure 14b indicates changes in FRAP over 6 weeks of cold storage. After 6 weeks, treatment GA + LM + PP and uncoated control recorded the highest (1111.16 mM TE/L PJ) and lowest (1062.23 mM TE/L PJ) FRAP values. However, there was no significant difference between FRAP values recorded for uncoated fruit and GA + LM + PP coating. After 6 weeks, the hierarchy for antioxidant capacity with respect to FRAP was GA + LM + PP > GA > Control > GA + PP > GA + LM. Overall, despite the noticeable changes in RSA, there was no significant difference between uncoated and coated fruit for large parts of the storage period.

#### 3.5.3. ABTS^+^ Scavenging Activity

Figure 14c shows the change in RSA evaluated with ABTS^+^ assay of coated and uncoated pomegranates over 6 weeks of storage. From day 0 to 6 weeks, scavenging activity values determined by ABTS+ assay ranged from 2274.75 to 2854.08 (µmol TE/mL PJ) for coated fruit and from 2305.53 to 2685.72 (µmol TE/L PJ) for uncoated fruit. After the 6-week storage period, treatments GA + LM and GA + LM + PP recorded the highest (2854.08 µmol TE/L PJ) and lowest (2376.14 µmol TE/L PJ) scavenging activity, respectively. However, there was no significant difference in scavenging activity between fruit coated with GA + LM (2854.08 µmol TE/L PJ) and uncoated fruit (2685.72 µmol TE/L PJ). 

## 4. Discussion

### 4.1. Physiological Response

#### 4.1.1. Weight Loss and Peel Microstructure

Yaman and Bayoindirli [77] explained that vapor-phase diffusion driven by a water vapor pressure gradient and respiration primarily causes weight reduction in fresh fruit and vegetables. In this present study, retarded weight loss recorded for fruit coated with GA coating formulations could be ascribed to the effect of coatings as semi-permeable barrier against gases, moisture and solute movement [78,79]. Ali et al. [78] demonstrated that different concentrations (5–20% *w/v*) of GA coatings retarded weight loss in ‘Money Maker’ tomato during 20 d of storage (20 °C), with a higher concentration of GA recording the least weight loss. Despite being natural hydrophilic biopolymers [80,81], GA coatings provided additional benefits in reducing weight loss compared to uncoated fruit packaged with Xtend^®^ liners only. Similarly, the reduction of weight loss by GA coatings with or without active components at different concentrations has been reported in fresh fruit such as pomegranate [58,61], papaya [48], green chilies [82] and banana [83].

The SEM images revealed that weight loss reduction in coated fruit could be attributed to coatings forming a layer that inhibits loss of moisture via lenticels [84]. Maqbool et al. [85] similarly reported that coatings reduced weight loss in ‘Pisang Berangan’ banana stored for 28 d at 13 °C (followed 5 d at 25 °C) by completely covering the cuticles and blocking the surface openings. The addition of either LM and/or PP to GA coatings appeared to enhance resistance to weight loss in fruit samples between 4 and 6 weeks. In addition, since LM consist mostly of volatile terpenoids and non-terpenoid constituents and not lipids, the contribution of LM to weight loss reduction may be minimal [86,87]. However, some authors have outlined that both LM and PP, when added to coatings, may contribute to weight loss reduction because of their lipophilic nature, which possibly reduces gas diffusion and losses in weight due to the respiratory process [44,88].

#### 4.1.2. Respiration Rate

Meighani et al. [58] linked higher water loss in uncoated ‘Malase Torshe Saveh’ pomegranates with an increase in R_CO2_ over 120 d of cold storage (4.5 ± 0.5 °C). Furthermore, as storage duration was extended, peel porosity increased, which explained higher R_CO2_ in uncoated fruit. Application of coatings as semi-permeable barriers on the surface of fresh fruit can modify the internal atmosphere and the concentration of CO_2_ and O_2_ [30,89]. In this present study, higher R_CO2_ was recorded for uncoated control compared to coated pomegranates at every storage period. The different GA coating formulations (GA, GA + LM, GA + PP and GA + LM + PP) showed great potential to reduce R_CO2_, at 2, 4 and 6 weeks of storage. This observation can be attributed to lesser gas interchange and lower oxygen availability for the respiration process due to the application of coatings [90,91]. These findings corroborate with Chen et al. [92], who found that application of coatings delayed the rise in R_CO2_ of ‘Nanfeng’ mandarin during 100-day storage period at 6 °C. Moreover, the application of coatings has been shown to lower R_CO2_ during the storage of fruit types such as tomato [25], sweet cherry [93], plum [94], banana [85] and others. The effect of adding LM or PP to GA coatings on RCO_2_ pattern was not clear; however, previous studies suggested that LM applications resulted in minor oxygen consumption and CO_2_ production due to reduced microbial growth caused by active compounds in the essential oil [95]. Azarakhsh et al. [44] also demonstrated that the addition of LM (0.1–0.3%) to sodium alginate coatings significantly reduced R_CO2_ of fresh-cut apples because of the lipophilic nature of LM, which possibly reduced gas diffusion. Likewise, some authors have reported that PP exhibits lipophilic properties, which improve barrier properties of coatings and thereby minimizing gas diffusion [88]. 

### 4.2. Decay Incidence and Fruit Internal Decay

The resistance to decay incidence and internal decay exhibited by ‘Wonderful’ pomegranates coated with GA + LM and GA + PP corroborates with recent studies which have proven the antimicrobial activities against postharvest pathogens of either LM or PP alone and when combined with coatings [60,96]. In this present study, internal decay observed was due to grey mold (*Botrytis cinerea*) and blue mold (*Penicillium* spp.) [64,97,98]. Treatments GA, GA + LM and GA + PP appeared to minimize the incidence of decay. In previous related studies, LM at different concentrations (0, 10, 50 or 100 g L^−1^) added to coatings improved resistance against postharvest fungal pathogens linked with pomegranate fruit [96]. Polyphenolic compounds from PP have been reported to exhibit antimicrobial activity against a broad spectrum of fungi [99]. The synergic effect of components such as tannins, punicalagin, castalagin, catechin and gallocatechin has been implicated in antifungal activity of PP [100]. Therefore, minimal development of decay in treatments GA + PP and GA + LM + PP could also result from the PP contribution in reducing fungi proliferation. 

### 4.3. Physicochemical and Textural Properties

#### 4.3.1. Color

The color of pomegranate is an essential attribute affecting marketability, purchasability and consumer preference [68,69,101]. For L* values, the findings corroborated with Meighani et al. [58] who observed no significant differences between coated and uncoated ‘Malase Torshe Saveh’ pomegranates during cold storage (4.5 ± 0.5 °C and 90 ± 5% RH). Contrary, research work reported by Selcuk and Erkan [102] attributed the decline in peel lightness to increased weight loss in uncoated ‘Hicaznar’ pomegranate when compared to fruit packed in MAP polyliners.

For a* values, the results agreed with Fawole et al. [103], who reported no significant changes for Ruby’ pomegranate arils stored at 2 or 5 °C for 16 weeks. However, the slight initial increase in redness for GA + LM treatment (day 0 to 2 weeks) could be attributed to synthesis of anthocyanin pigments as a response to cold temperature in the first few weeks of storage [104,105,106]. Cold storage temperatures have been reported to favor the biosynthesis of anthocyanins in fruit, which often results in peel color maintenance [107]. The reduction of anthocyanin degradation occurs by inhibiting polyphenoloxidase activity through the modification of the internal fruit atmosphere by surface coatings [108]. The C* value measure the degree of saturation of color and is proportional to the strength of the color [68,69]. There was minimal evolution in saturation of color across all coating treatments and no significant changes in hue-angle with the extension of storage duration.

#### 4.3.2. Total Soluble Solids and Titratable Acidity

The effect of coating treatments on TSS was not significant despite some notable changes. However, between 0 and 6 weeks, GA coating treatments recorded lower TSS values than uncoated fruit. Application of coating treatments has been reported to reduce changes in TSS by inhibiting respiration processes leading to conversion of starch to sugar during storage [109,110]. Previous studies have reported that organic acids of fruit are substrates consumed during storage in respiratory processes [77,111,112]. Edible coatings form semipermeable barriers, which reduce movement of gases thereby limiting respiratory and metabolic process of the fruit, reducing the loss of nutritional components such as organic acids [68,113,114]. Therefore, the application of coatings often reduces the loss of TA content during cold storage. In this present study, coating treatments (GA, GA + LM, GA + PP and GA + LM + PP) recorded higher TA content than uncoated fruit.

#### 4.3.3. TSS/TA and Brim A

The TSS/TA ratio is a reliable indicator of fruit maturity linked to consumer organoleptic perception of sweet and sour taste [115,116]. The lower TA content in coated fruit could have played a major role in keeping TSS/TA higher than uncoated fruit. The retention of higher TA content can be linked to lower cellular activity in the fruit, in which organic acids serve as substrates in the Krebs cycle to gain energy for repairing the aging cell and membranes [117,118]. The results from this present study corroborate with Zaouay et al. (106), who observed a strong relationship between TA and the ratio of TSS: TA for a comparison study between several pomegranate cultivars on juice yield, quality parameters and phytochemicals. The BrimA allows for small amounts of acid than sugar to make the same numerical changes to BrimA index [119]. Similar to this study, a strong association between BrimA and TSS has been reported for pomegranate fruit during cold storage, indicating that changes in BrimA were mainly influenced by TSS [70].

### 4.4. Phytochemicals and Antioxidant Properties

#### 4.4.1. Phytochemicals

##### Total Phenolic Content

The application of coatings in fresh fruit before cold storage is often related to increased production of phenolic compounds as a response to low temperature and change in the internal atmosphere [47,120,121]. However, in this present study, there were no significant differences between the TPC of coated and uncoated fruit. Similar results were reported by Meighani et al. [58], who noted that within 40 d of cold storage at 4.5 °C (followed by 3 d at 20 °C), there were no significant differences between the TPC coated and uncoated ‘Malase Torshe Saveh’ pomegranates. However, beyond 40 d, the TPC content in uncoated pomegranates declined and remained lower than coated fruit. Therefore, this suggests that changes in TPC could have clearly been extended beyond 6 weeks. Authors have linked changes in phenolic compound synthesis and biosynthesis to phenylalanine ammonia-lyase and polyphenoloxidase enzymes, respectively, which can be influenced by the application of coatings on the fruit surface through inhibition of gas exchange and limiting moisture loss [122,123,124]. 

##### Total Anthocyanin Content

The increase in TAC observed for uncoated and coated fruit corroborates with studies that report that low temperatures, usually under cold storage, favor the enzyme mediated biosynthesis of anthocyanins in pomegranate fruit [107]. Different authors have observed continued accumulation of anthocyanins induced by cold temperature (5–10 °C), followed by a sharp decline as extended storage duration [107,125]. The application of GA coatings proved beneficial in minimizing loss of TAC as storage duration was extended from day 0 to 6 weeks. Interrelated results were reported by Varasteh et al. [105], who demonstrated that application coatings on ‘Rabbab-e-Neyriz’ pomegranates during cold storage (2 ± 0.5 °C) for 45 d (followed by 3 d of shelf life at 20 °C) reduced degradation of anthocyanins and color deterioration. The results agreed with Tezotto-Uliana et al. [126], who similarly observed a reduction in loss of anthocyanins during cold storage of coated ‘Autumn Bliss’ raspberries. Earlier studies reported by Zhang and Quantick [127] also showed that chitosan (1 and 2%) coatings applications on fresh strawberries and raspberries during 16 d of cold storage (4 °C) reduced of loss of anthocyanin content. The direct application of LM has been reported to increase anthocyanin content in fruit tissues of strawberries [128] and blueberries [129]. 

##### Total Flavonoid Content

In the present study, the TF content of coated and uncoated fruit increased as storage duration was extended. Furthermore, fruit coated with GA coating formulations recorded higher TF content than uncoated as the storage period increased. Similarly, Meighani et al. [58] observed an initial increase in TF content of coated and uncoated ‘Malase Torshe Saveh’ pomegranates during 40 d of cold storage (4.5 ± 0.5 °C with 95% RH). Varasteh et al. [105] linked increase in TFC in ‘Rabbab-e-Neyriz’ pomegranate with initial biosynthesis of flavonoids as a defense mechanism in the first days after harvest presumably as a reaction to stress [130]. The application of coating treatments restricted the conversion of flavonoids into secondary phenolic compounds due to the ripening process or enzyme mediated depletion during cold storage [131]. 

##### Ascorbic Acid Content

The decrease in ascorbic acid content during cold storage is usually associated with increased rate of senescence [132]. In the present study, ascorbic acid content of fruit varied as storage duration was extended, with coated fruit recording the highest values at every storage period. However, for large periods during storage, the difference between some coated and uncoated fruit was not significant. The results corroborate with Mantilla et al. [133], who reported the ineffectiveness of multi-layered edible coating of sodium alginate, pectin and calcium chloride to inhibit loss of ascorbic acid of fresh-cut apples during cold storage (4 °C for 15 d). In contrast, Gonzalez-Aguilar et al. [134] outlined that edible coatings modify the internal atmosphere of fresh produce, which promotes accumulation of secondary metabolites and ascorbic acid during cold storage. Likewise, Ehteshami et al. [135] found that application of CH (2% *w/v*) coatings enriched with organic acids on ‘Rabbab-e-Neyriz’ pomegranate resulted in higher ascorbic acid content during 102 d of cold storage (2 °C). Based on the results in this study, application of coatings with or without LM and/or PP exerted a minimal effect on ascorbic acid during storage. 

#### 4.4.2. Antioxidant Properties

The presence and production of ascorbic acid and phenolic compounds, including flavonoids, tannins and phenolic acids, plays an important role in the induction of RSA [136,137]. Anthocyanins are the most important group of flavonoids in pomegranate and often related to antioxidant capacity [76,104]. Generally, coating applications are known to improve the antioxidant activity of fresh produce by limiting the loss of phytochemicals such as phenolics and ascorbic acid. Moreover, application of coating treatments can modify internal atmosphere of fresh produce, which promotes accumulation of secondary metabolites and ascorbic acid during cold storage [134]. Similarly, packaging pomegranates in plastic liners have also been reported to improve antioxidant capacity and overall quality of the fruit during cold storage [14].

Using the DPPH assay, the slight increase in RSA between harvest and 4 weeks could be attributed to the effect of coatings and plastic liners in enhancing the production and improving antioxidant capacity. The subsequent decrease observed after 4 weeks could be attributed to many factors involving the enzyme mediated degradation of phenolic compounds related to the antioxidant system of aril juice, which often occurs when storage duration is extended in pomegranate [125,138]. However, other mechanisms may have been responsible for antioxidant activity of pomegranate fruit during cold storage after application of coating treatments.

According to Moniruzzaman et al. [139], FRAP method for determining antioxidant capacity is based on the reduction of Fe (III)-tripyridyltriazine complex to Fe (II)-tripyridyltriazine at low pH by electron-donating antioxidants. After 6 weeks, higher antioxidant capacity for treatments such as GA and GA + LM + PP than uncoated fruit suggests a higher accumulation of active compounds such as anthocyanins, flavonoids and ascorbic acid [91]. However, the effect of coating application of RSA as quantified by the FRAP and ABTS assays was unclear and could not be related to phytochemical changes. Overall, using the DPPH assay, the effect of coatings was more pronounced after 4 weeks of storage, whilst the pattern of change was not clear using FRAP and ABTS assays. The assays DPPH, FRAP and ABTS+ have shown variability when determining antioxidant capacity [140].

## 5. Conclusions

Since pomegranate fruit has high nutritional and economic value, control strategies to minimize postharvest quality loss along the supply chain are a research priority. The outcomes from this study showed that gum arabic (1.5% *w/v*) coatings enriched with lemongrass oil (0.1% *v/v*) and/or pomegranate peel extract (1% *w/v*) are potential natural postharvest treatments to reduce weight loss, respiration rate and reduce decay incidence in pomegranate fruit. Gum arabic formulations effectively enhanced total flavonoids and anthocyanins as the storage period was extended. ‘Wonderful’ pomegranate fruit are currently packaged in micro-perforated (20 µmm) Xtend^®^ liners and marketed on a weight basis, and visual appearance, the coatings formulated in this study could prove important in minimizing postharvest losses and ensuring the availability of the fruit on the consumer market. Future studies should optimize gum arabic coatings with available bioactive compounds to improve the physicochemical, phytochemical and antioxidant properties and study the effects of coatings on organoleptic properties of coated pomegranates and their potential toxicity. The study also showed the uniqueness in the response of pomegranate compared to other fruit types to the application of the selected coatings, highlighting the necessity for fruit-specific coatings for the best packaging and fruit quality outcomes.

## Data Availability

Not applicable.
